# Molecular Characterization of Ovarian Endometriosis in Saudi Arabian Women: Insights into Inflammatory, Autophagic, and Epigenetic Dysregulation

**DOI:** 10.3390/ijms27104598

**Published:** 2026-05-20

**Authors:** Saber Nahdi, Maria Arafah, Felice Petraglia, Maroua Jalouli, Abdullah Alamri, Mohammad Alanazi, Md Ataur Rahman, Saleh Alwasel, Abdel Halim Harrath

**Affiliations:** 1 Department of Zoology, College of Science, King Saud University, Riyadh 11451, Saudi Arabia; nehdisabeur@gmail.com (S.N.); salwasel@ksu.edu.sa (S.A.); 2 Department of Pathology, College of Medicine, King Saud University, Riyadh 11451, Saudi Arabia; marafah83@gmail.com; 3 Department of Experimental, Clinical and Biomedical Sciences “Mario Serio”, University of Florence, 50134 Florence, Italy; felice.petraglia@unifi.it; 4 Department of Biology, College of Science, Imam Mohammad Ibn Saud Islamic University (IMSIU), Riyadh 11623, Saudi Arabia; mejalouli@imamu.edu.sa; 5 Department of Biochemistry, College of Science, King Saud University, Riyadh 11451, Saudi Arabia; abdullah@ksu.edu.sa (A.A.);; 6 Department of Neurology, University of Michigan, Ann Arbor, MI 48109, USA; ataur1981rahman@hotmail.com

**Keywords:** endometriosis, ovary, inflammation, autophagy, epigenetic, Saudi patients

## Abstract

Ovarian endometriosis (OE) is a chronic, inflammatory gynecological disorder associated with sterility and an elevated risk of ovarian cancer. Despite its high prevalence, the complex molecular mechanisms governing OE pathogenesis remain poorly investigated. We conducted a comprehensive histopathological and molecular investigation of OE in a cohort of 188 Saudi women (88 patients with OE and 100 healthy controls) using histopathological, qRT-PCR, immunostaining, and Western blot techniques. Histopathological analysis confirmed significant stromal fibrosis and chronic inflammation in endometriotic lesions. Gene expression profiling revealed a pro-proliferative, anti-apoptotic signature, marked by the upregulation of *PTTG1* and the downregulation of *TNFRSF10D*, *CDK4*, and *CDKN1A*. Interestingly, we identified a post-transcriptional regulatory paradox in the inflammatory response: while *IL-6* mRNA was significantly upregulated, its corresponding protein level was downregulated, suggesting a novel, tightly controlled mechanism to limit excessive local inflammation. Besides the increased autophagic activity and decreased Ubiquitin mRNA levels, epigenetic dysregulation was prominent, characterized by the upregulation of DNA methyltransferase DNMT3B and the downregulation of the histone variant H3.1. These findings elucidate novel molecular pathways underlying OE pathogenesis as evidenced by a post-transcriptional paradox in IL-6 expression, and uncover key dysregulations spanning cell proliferation, apoptosis, inflammation, autophagy, and epigenetic regulation.

## 1. Introduction

Ovarian endometriosis (OMA) is a gynecological condition characterized by the growth of endometrial tissue into the ovary (Matias-Guiu & Stewart). It is associated with pelvic pain, risk of developing ovarian cancer, and infertility [1,2]. The pathophysiology of endometriosis is intricate and involves genetic, epigenetic, inflammatory, and hormonal influences [3,4]. It is well described that ectopic endometrial tissues exhibit abnormal responses to hormonal signals, altered immune function, aberrant cell proliferation, and impaired apoptosis [5]. Several studies have linked alterations in inflammatory markers, disrupted intrafollicular hormone environments, and abnormal intrafollicular cytokines to endometriosis and infertility [6]. The pro-inflammatory cytokine interleukin-6 (IL-6) is a factor in the molecular pathways involved in the etiology of endometriosis, where it promotes an inflammatory response and angiogenesis and enhances cell survival, contributing to the persistence and progression of endometriotic tissue [7]. Women diagnosed with endometriosis frequently experience infertility attributed to various factors, such as altered ovarian function, impaired implantation, and tubal dysfunction [8]. In this context, IL-6 may significantly contribute to endometriosis-associated infertility, potentially through its effects on autophagy [9]. In fact, the latter is a crucial mechanism for preserving cellular homeostasis [10], and disruption in autophagy mechanisms has been implicated in several pathological conditions, including cancer and autoimmune diseases [11]. In endometriosis, autophagic mechanisms may facilitate the survival of ectopic endometrial cells and contribute to disease persistence [12].

In addition to inflammation and autophagy, epigenetic factors are increasingly being acknowledged as significant contributors to the spread and progression of endometriosis [13]. DNMT3B, a DNA methyltransferase, is an important epigenetic regulator of endometriosis [14]. It participates in DNA methylation, resulting in gene silencing and chromatin remodeling [15]. Alterations in DNMT3B expression may play a role in the aberrant gene expression patterns observed in ovarian endometriosis, particularly those associated with inflammation and immune modulation [16].

Previous studies have reported that the prevalence of endometriosis varies across racial and ethnic groups [17]. Notably, among women diagnosed with endometriosis, Black and Hispanic women exhibit a lower prevalence and tend to receive diagnoses at older ages compared with White women [18]. As these disparities may reflect diagnostic biases associated with race and ethnicity, the present study aimed to investigate the occurrence of endometriosis in Saudi patients, with a particular focus on the roles of interleukin-6 (IL-6), autophagy, and epigenetic factors in disease pathogenesis. In our recent bioinformatics-based analysis, we demonstrated that IL-6, microRNAs, and autophagy pathways interact to regulate inflammation and cellular repair mechanisms in ovarian disorders, including ovarian endometriosis [19]. A deeper understanding of how racial and ethnic backgrounds influence disease presentation may help tailor clinical approaches for individual patients—from initial suspicion through diagnosis and treatment—and enhance the accuracy and timeliness of endometriosis diagnosis.

## 2. Results

### 2.1. Histopathological Comparison Between Normal and Ovarian Endometriosis Tissues

**Figure 1:**
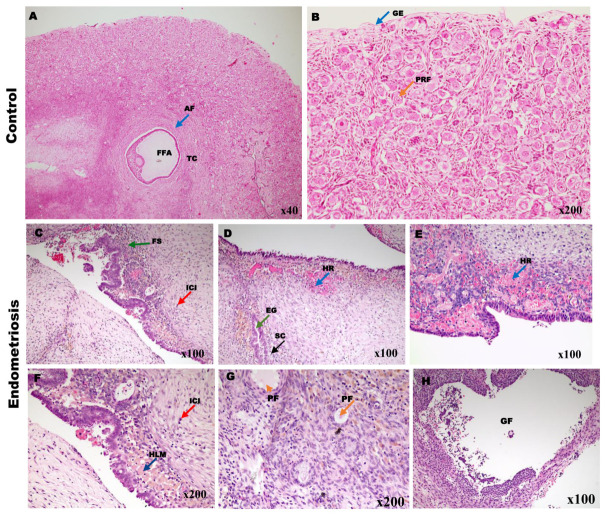
Representative histological sections of ovarian tissue stained with Hematoxylin and Eosin (H&E). The figure contrasts normal ovarian morphology with the pathological alteration characteristics of ovarian endometriosis. Control Tissue ((**A**,**B**)): (**A**) is a low-magnification view of the control ovarian cortex, displaying normal architecture, including a developing antral follicle (AF). Panel (**B**) is a high-magnification view of the control cortex, showing the normal density and morphology of primordial follicles (PRF). Ovarian Endometriosis (Panels (**C**–**H**)): Panels (**C**,**D**) illustrate the characteristic features of ovarian endometriosis, including ectopic Endometrial Glands (EG) ((**D**), green arrow) and surrounding Stromal Cells (SC) ((**D**), black arrow), embedded within a dense Fibrotic Stroma (FS) ((**C**), green arrow). Note the presence of significant Inflammatory Cell Infiltration (ICI) ((**F**), red arrow) adjacent to the lesion. (**E**) shows a section of the endometriotic lesion with evidence of hemorrhagic regions consistent with recent or prior bleeding and tissue breakdown, characterized by a Hemorrhagic Region (HR). (**F**) highlights the inflammatory response within the endometriotic stroma, showing Inflammatory Cell Infiltration (ICI) and the presence of Hemosiderin-Laden Macrophages (HLM) (FIG F blue arrow), indicative of chronic hemorrhage. (**G**) demonstrates a Primordial Follicle (PF) in proximity to the endometriotic lesion, illustrating the potential impact of the disease on the presence and proximity of follicular structures (e.g., primary and Graafian follicles). Finally, (**H**) shows a large Graafian Follicle (GF) adjacent to the endometriotic tissue, suggesting the presence of advanced follicular stages despite the surrounding pathology. These histological findings confirm pathological alterations in ovarian endometriosis, including fibrosis, chronic inflammation, and hemorrhage, which may contribute to pain, infertility, and ovarian dysfunction. AF: Antral Follicle, FFA: Fluid-Filled Antrum, TC: Theca Cells, GE: Germinal Epithelium, FS: fibrotic stroma, ICI: inflammatory cell infiltration, HR: hemorrhagic region, EG: endometrial gland, SC: stromal cells, PF: primordial follicle; PRF: primary follicle, GF: Graafian follicle, HLM: hemosiderin-laden macrophage.

The histological architecture of normal ovarian tissues (Figure 1A,B) was well-preserved, featuring normal epithelium and organized stromal and cortical components. Conversely, ovarian endometriosis tissues (Figure 1C–H) exhibited pathological characteristics, including ectopic endometrial glands, stromal cells with significant stromal fibrosis, and persistent inflammatory cell infiltration (Figure 1C,F, red arrow). Moreover, hemosiderin-laden macrophages (Figure 1F, blue arrow) were commonly found, signifying recurrent hemorrhage (Figure 1D blue arrow), a characteristic hallmark of endometriotic lesions. The combination of endometriotic lesions and ovarian follicles (Figure 1G,H) directly affected follicular integrity. The development of primary (Figure 1G) and Graafian follicles (Figure 1H) in endometriotic lesions raises concerns regarding diminished ovarian reserve and function. The inflammatory microenvironment, characterized by substantial immune cell infiltration and fibrosis (Figure 1C, red and green arrow) may hinder folliculogenesis and diminish ovarian responsiveness.

### 2.2. Real-Time PCR-Based Array Analysis and Gene Dysregulation in Ovarian Endometriosis

**Figure 2:**
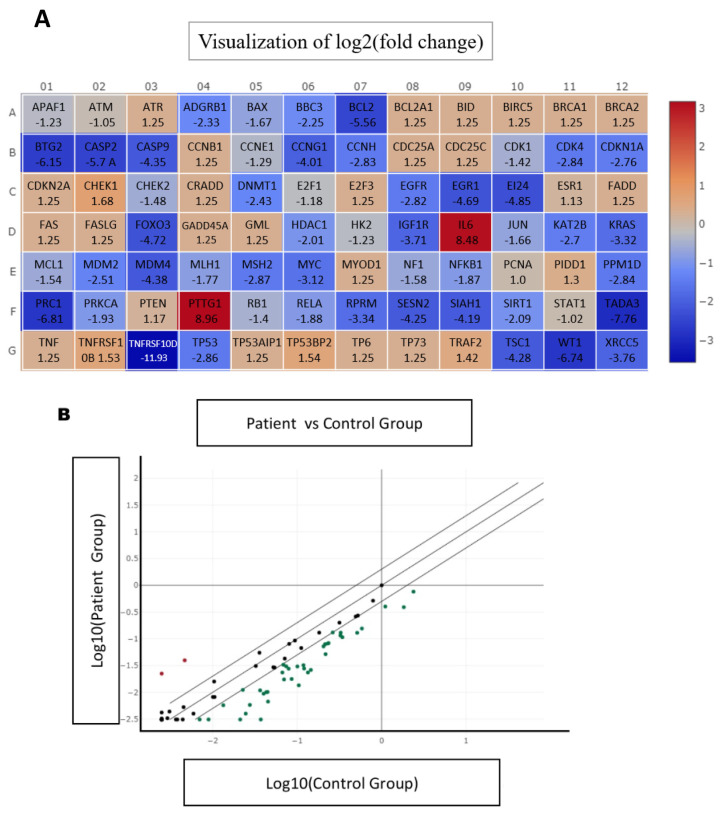
Differential gene expression profile in endometriosis determined by quantitative real-time PCR array analysis. (**A**) Hierarchical Clustering Heatmap. The heatmap illustrates the differential expression patterns of the 84 analyzed genes across all individual samples. The color intensity represents the Z-score of the log2 (fold change), where red indicates upregulated expression and blue indicates downregulated expression in the endometriosis group relative to the control group. (**B**) Scatter Plot Analysis of Gene Dysregulation. A scatter plot analysis was conducted, with the central line representing genes exhibiting no change in expression. Genes identified as upregulated are indicated by red dots, while green dots denote downregulated genes.

A real-time PCR-based gene expression array was used to examine the molecular alterations linked to ovarian endometriosis, focusing on the mRNA levels of 84 genes associated with a particular biological pathway. Heatmap analysis (Figure 2A) demonstrated significant differential gene expression between normal ovarian tissues and ovarian endometriotic lesions, suggesting extensive transcriptional reprogramming. Figure 2B presents a scatter plot illustrating these changes, with the upregulated genes indicated in red and the downregulated genes indicated in green. PTTG1 and IL6 were among the most significantly upregulated genes, exhibiting fold changes of +8.77 and +8.48.

Moreover, several essential genes associated with cell cycle regulation, apoptosis, and epigenetic modifications were significantly downregulated, in particular TNFRSF10D (tumor necrosis factor receptor superfamily member 10D), CDK4 (cyclin-dependent kinase 4), and CDKN1A (cyclin-dependent kinase inhibitor 1A), as well as DNMT1 (DNA methyltransferase 1). These findings collectively highlight the intricate relationship between inflammation, cell cycle dysregulation, and epigenetic modifications in the pathophysiology of ovarian endometriosis.

### 2.3. Dysregulated Gene Expression Profile in Ovarian Endometriosis

**Figure 3:**
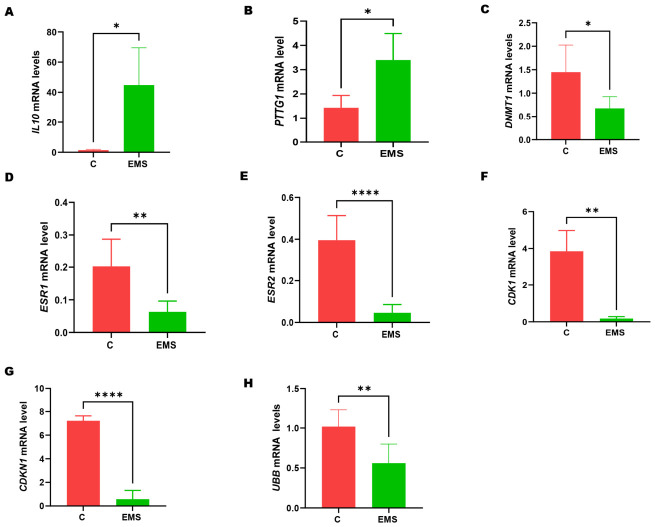
Differential gene expressions in ovarian endometriosis by quantitative real-time PCR (qRT-PCR). The expression levels of eight selected genes (*IL-10*, *PTTG1*, *DNMT1*, *ESR1*, *ESR2*, *CDK1*, *CDKN1A*, and *UBB*) in ovarian endometriosis tissue compared to control ovarian tissue show significant differential expression between the two groups. (**A**,**B**) show the relative expression of the significantly upregulated genes, *IL-10* and *PTTG1*, respectively. (**C**–**H**) display the relative expression of the significantly downregulated genes: *DNMT1*, *ESR1*, *ESR2*, *CDK1*, *CDKN1A*, and *UBB*. Data are presented as mean ± SD. Statistical significance was determined by unpaired two-tailed Student’s *t*-test (* *p* < 0.05, ** *p* < 0.01, **** *p* < 0.0001).

The key genes involved in the molecular mechanisms underlying ovarian endometriosis, including IL10, UBB, DNMT1, ESR1, ESR2, CDK1, CDKN1A, and PTTG1, were analyzed (Figure 3). Results showed that the mRNA of IL-10 and PTTG1 genes were significantly increased (Figure 3A,B). In contrast, UBB, DNMT1, ESR1, ESR2, CDK1, and CDKN1A were notably downregulated (Figure 3C–H). Ubiquitin B (UBB) reduced expression indicates a potential impairment of proteasomal function, which may result in the accumulation of cellular stress. The notable decrease in ESR1 (estrogen receptor alpha) and ESR2 (estrogen receptor beta) expression suggests a potential disruption in estrogen signaling, which is essential for the hormonal regulation of endometrial tissue. The suppression of CDK1 (cyclin-dependent kinase 1) and CDKN1A (cyclin-dependent kinase inhibitor 1A) expressions indicates a disruption in cell cycle regulation, which may lead to abnormal cellular proliferation and the survival of endometriotic lesions.

### 2.4. Dysregulation of IL-6 in Ovarian Endometriosis: Upregulated mRNA but Downregulated Protein Expression

**Figure 4:**
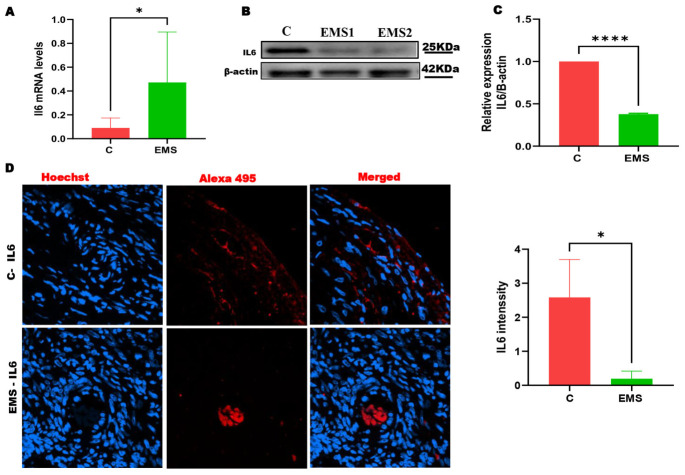
Protein and mRNA expression of the pro-inflammatory cytokine IL-6 in the ovarian endometriosis and control groups. (**A**) qRT-PCR of IL-6 mRNA levels. (**B**) Representative immunoblots. β-actin was used as internal control. (**C**) Densitometric quantification of IL-6 protein levels in ovarian endometriosis and control groups. (**D**) Immunofluorescence staining for IL-6 protein expression (X40). The results demonstrate a notable reduction in IL-6 protein expression in the endometriosis group compared to the control group. However, IL-6 mRNA levels were significantly upregulated in endometriotic tissues, suggesting a post-transcriptional or translational regulatory mechanism affecting IL-6 protein synthesis. Data are presented as mean ± SD. Statistical significance was determined by unpaired two-tailed Student’s *t*-test (* *p* < 0.05, **** *p* < 0.0001).

We performed analyses of the mRNA and protein expression of IL-6 in ovarian endometriosis. Quantitative real-time PCR (qRT-PCR) analysis demonstrated a significant increase in IL-6 mRNA levels in endometriotic tissues relative to normal ovarian tissues, indicating increased transcriptional activity (Figure 4A). Protein expression analysis using Western blotting (Figure 4B,C) and immunofluorescence (Figure 4D) techniques revealed a downregulation of IL-6 protein levels in endometriotic lesions compared to control, indicating a clear discordance between transcriptional and translational outputs.

### 2.5. Enhanced Autophagic Activity in Ovarian Endometriosis: Upregulation of LC3 at Both mRNA and Protein Levels

**Figure 5:**
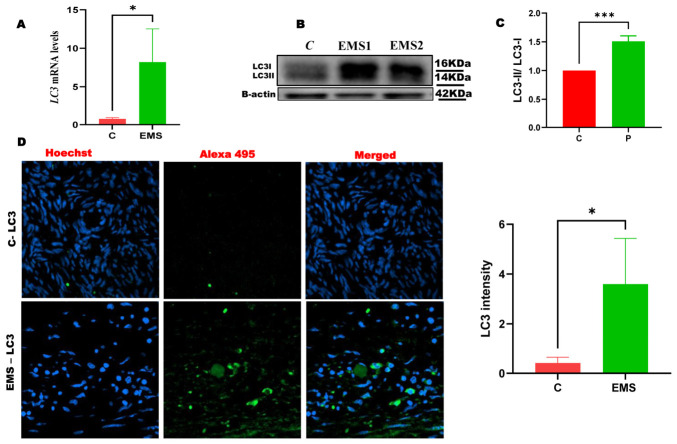
Protein and mRNA expression of the autophagic marker LC3 in the ovarian endometriosis and control groups. (**A**) qRT-PCR of LC3 mRNA levels. (**B**) Representative immunoblots. (**C**) Densitometric quantification of LC3 protein levels in the ovarian endometriosis and control groups. β-actin was used as internal control. (**D**) Immunofluorescence staining for LC3 protein expression (X40). The results demonstrate a notable increase in LC3 protein and mRNA levels in the endometriosis group compared to the normal group. Data are presented as mean ± SD. Statistical significance was determined by unpaired two-tailed Student’s *t*-test (* *p* < 0.05, *** *p* < 0.001).

To determine the extent of autophagic activity in ovarian endometriosis, we analyzed the transcriptional and protein levels of the key autophagic marker, microtubule-associated protein 1A/1B-light chain 3 (LC3). qRT-PCR analysis demonstrated a significant increase in LC3 mRNA levels in endometriotic tissues relative to those in the control group (Figure 5A). Protein expression analysis using Western blotting (Figure 5B,C) and immunofluorescence techniques (Figure 5D) consistently showed a notable increase in LC3 protein levels in ovarian endometriotic lesions. The concurrent upregulation of both mRNA and protein levels indicates increased autophagy-related marker expression in endometriotic tissues compared to control groups.

### 2.6. Differential Expression of DNMT3B and H3.1 in Ovarian Endometriosis

**Figure 6:**
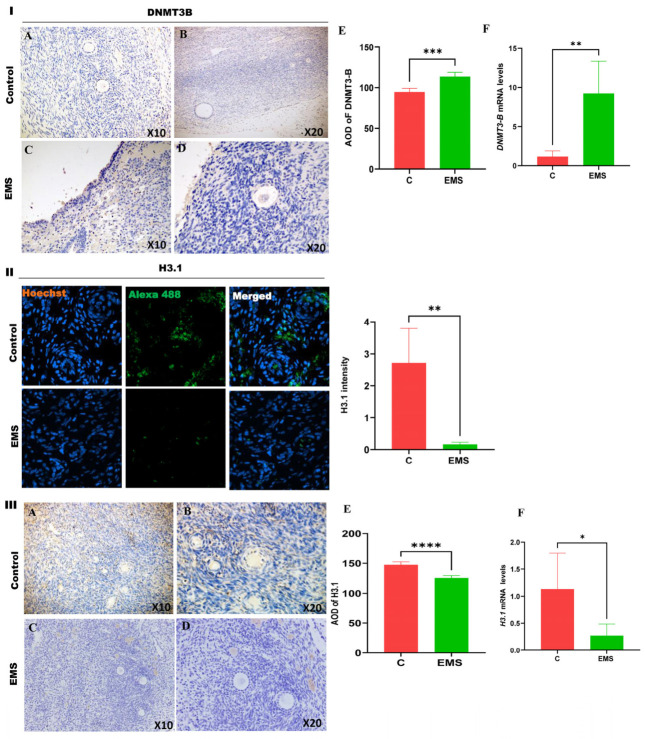
Differential expression of epigenetic regulators DNMT3B and histone H3.1 in ovarian endometriosis. (**I**) Representative immunohistochemical (IHC) analysis of DNMT3B expression in control and ovarian endometriosis tissues. Normal ovarian tissue exhibits intact stromal architecture characterized by a dense, spindle-shaped cellular matrix and absence of ectopic endometrial glands or stroma (**A**,**B**). In contrast, ovarian endometriotic lesions display a marked increase in DNMT3B nuclear immunoreactivity (**C**–**E**). Semi-quantitative analysis of DNMT3B protein expression, presented as average optical density (AOD), confirms a significant elevation in endometriosis tissues relative to controls. Consistently, quantitative RT-PCR analysis reveals a pronounced upregulation of DNMT3B mRNA levels in the endometriosis group (**F**). These findings suggest that DNMT3B overexpression may contribute to epigenetic reprogramming in ovarian endometriosis through aberrant DNA methylation and altered gene regulation. (**II**) Representative immunofluorescence staining of histone H3.1 in ovarian tissues. H3.1 exhibits strong nuclear localization in control samples, whereas a marked reduction in fluorescence intensity is observed in ovarian endometriosis tissues, indicating diminished H3.1 protein abundance (X40). (**III**) Representative IHC and quantitative RT-PCR analyses of histone H3.1 expression. Ovarian endometriosis tissues show a significant decrease in H3.1 protein expression (**A**–**E**), accompanied by a parallel reduction in H3.1 mRNA levels (**F**), compared with control tissues. Collectively, these data indicate that H3.1 dysregulation is a prominent feature of ovarian endometriosis and may play a critical role in disease-associated chromatin remodeling and epigenetic instability. Data are presented as mean ± SD. Statistical significance was determined by unpaired two-tailed Student’s *t*-test (* *p* < 0.05, ** *p* < 0.01, *** *p* < 0.001, ****, *p* < 0.0001).

We also examined the expression of DNMT3B (DNA methyltransferase 3-beta), a key enzyme involved in de novo DNA methylation, and H3.1 (Histone H3.1), a core histone protein linked to chromatin organization. Our findings indicated a notable upregulation of DNMT3B at both the transcriptional and protein levels, as shown in Figure 6I(A,B). In contrast, H3.1 expression was markedly downregulated in endometriotic lesions, as demonstrated by Immunofluorescence, immunohistochemistry and qRT-PCR (Figure 6II,III).

## 3. Discussion

This study investigated histopathological and molecular changes in ovarian endometriosis in Saudi patients by analyzing differential gene expression, inflammatory responses, epigenetic modifications, and autophagic activity. The findings offer novel insights into the pathophysiology of ovarian endometriosis and help identify potential therapeutic targets for disease management.

Histopathological analysis using H&E staining revealed notable architectural distinctions between normal ovarian tissues and endometriotic lesions (Figure 1). Normal ovarian tissues displayed a well-preserved epithelium, organized stromal components, and intact follicular structures. In contrast, ovarian endometriotic lesions present with ectopic endometrial glands, significant stromal fibrosis, persistent inflammatory infiltration, and hemosiderin-laden macrophages [20]. These pathological characteristics highlight the inflammatory and fibrotic aspects of ovarian endometriosis, which may lead to impaired folliculogenesis and ovarian dysfunction [21,22], and an increased risk of ovarian cancer [2]. In agreement with prior studies, follicular defects, such as abnormal follicular growth and decreased follicular size and oocyte quality, are observed in women with endometriosis [23]. Clinical evidence from in vitro fertilization and oocyte donation programs suggested that patients with endometriosis may experience decreased follicular development [24,25]. Additionally, patients with endometriosis may have a deficiency in granulosa cell steroidogenesis, contributing to decreased oocyte function and reduced fertility rates [24].

Despite extensive investigations, the exact molecular mechanisms of ovarian endometriosis are not well understood. Our analysis using a real-time PCR-based gene expression array revealed significant transcriptional reprogramming in ovarian endometriotic lesions compared to normal ovarian tissues (Figure 2). The upregulated genes, such as PTTG1 and IL6, were related to increased cell proliferation and inflammation. Conversely, the downregulated genes, including TNFRSF10D, CDK4, CDKN1A, and DNMT1, are associated with disturbances in apoptosis, cell cycle regulation, and epigenetic control [26]. The overexpression of PTTG1 and IL6 corroborates previous research, indicating that these genes are pivotal in the pathogenesis of endometriosis through their roles in promoting inflammation and cellular proliferation [27]. Our further gene expression analysis indicated the significant upregulation of IL10 and PTTG1, whereas UBB, DNMT1, ESR1, ESR2, CDK1, and CDKN1A were downregulated in ovarian endometriotic tissues (Figure 3). The increased IL-10 expression, a cytokine that regulates immune responses, may indicate a compensatory anti-inflammatory response, whereas the downregulation of ESR1 and ESR2 indicates modified estrogen signaling, potentially leading to disrupted hormonal regulation in endometriosis [28]. The overexpression of PTTG1, a proto-oncogene and a crucial regulator of cell cycle, tumorigenesis, and other cellular processes, suggests a possible role in the abnormal cellular growth observed in endometriotic lesions. Collectively, these findings support earlier research demonstrating that dysregulation of estrogen receptors is a characteristic feature of endometriotic lesions [28,29].

Although the specific mechanism underlying the effect of endometriosis on fertility is unknown, research has indicated that an abnormal immune system may play a key role. Indeed, Harada and colleagues [30] found that infertile patients with endometriosis had considerably greater levels of IL-6 and TNF-α in their peritoneal fluid [30]. Thus, cytokines released by several cell types in the peritoneal fluid contribute to the development and progression of endometriosis and associated infertility. Interestingly, macrophages are thought to be the principal source of IL-6 in the peritoneal fluid [31,32,33]. This significant pro-inflammatory cytokine demonstrates a notable expression pattern in ovarian endometriosis [34]. In the current study, observed disparity between increased IL-6 mRNA and diminished protein levels indicates the participation of post-transcriptional regulation mechanisms in ovarian endometriosis. This regulation may encompass microRNA-mediated repression, modified mRNA stability, diminished translation efficiency, or increased proteasomal degradation. This contradictory trend may indicate a compensatory biological response designed to restrict excessive inflammatory signals in the local tissue milieu. Comparable mRNA–protein discrepancies have been documented in cytokine biology, reinforcing the likelihood of intricate regulatory mechanisms governing IL-6 expression. Further research is necessary to clarify the specific molecular processes governing this control and to confirm its functional importance in disease development.

One of these mechanisms is ubiquitination, which is involved in the breakdown of proteins and the control of several biological functions, including the cell cycle, DNA repair, and the stress response. Our analyses showed a significant decrease in the mRNA levels of the UBB gene in patients compared to controls (Figure 3H). In contrast, the expression of ubiquitin is increased during the secretory phase in both the glands and stroma of endometriotic tissues compared to controls [35]. Thus, despite having elevated mRNA levels, the downregulation of IL-6 protein expression may be due to a post-transcriptional mechanism, probably involving another mechanism rather than ubiquitination. Prior research has indicated comparable inconsistencies in cytokine expression, underscoring the intricate regulation of inflammatory mediators in endometriosis [7]. Nonetheless, further exploration of the post-transcriptional regulatory processes, including the study of miRNAs, in endometrial tissues is highly required [36].

Autophagy is essential for cellular homeostasis because it enables the degradation and recycling of damaged organelles and proteins [37]. Dysregulated autophagy plays an important role in the pathogenesis of endometriosis by enhancing cell survival and the persistence of ectopic endometrial cells [38]. Our findings revealed a notable upregulation of LC3 mRNA and protein levels in ovarian endometriotic tissues, as shown in Figure 5. Consistent with our results, many studies have reported the increased expression of autophagic biomarkers (Beclin-1 and LC3-II) in ovarian endometriomas compared to eutopic and normal endometria, supporting the idea of enhanced autophagic activity in endometrial tissues [39,40]. This enhanced autophagic activity potentially allows ectopic endometrial cells to endure unfavorable conditions, including oxidative stress and inflammation. This may lead to the proliferation, survival and persistence of ectopic endometrial cells by offering a mechanism for stress adaptation in hypoxic and inflammatory environments [41]. Hence, targeting autophagy-related pathways may serve as a new therapeutic approach for disrupting the survival mechanisms of endometriotic cells. Despite elevated amounts of LC3 mRNA and protein, this alone does not substantiate increased autophagic flux. Increased LC3 levels may indicate either enhanced autophagosome production or compromised degradation [42]. Additionally, p62/SQSTM1 is a key autophagy adaptor degraded during autophagic flux. Its accumulation indicates impaired degradation, while decreased levels suggest active autophagic flux, helping distinguish functional autophagy from lysosomal blockage [43]. Future investigations should use p62/SQSTM1 and flux tests to precisely differentiate between active autophagy and blocked autophagic degradation in endometriotic tissues.

Epigenetic modifications such as DNA methylation and histone alterations are essential for gene regulation and the development of various diseases [44]. DNMT1 is the most common methyltransferase in mammalian cells; it has an affinity for hemimethylated DNA and is localized near replication foci [45]. DNMT3A and DNMT3B, on the other hand, are typically involved in the development of DNA methylation patterns during embryonic development [46]. This study revealed a notable downregulation of DNMT1, upregulation of DNMT3B, and downregulation of H3.1 in ovarian endometriotic tissues (Figure 6). The upregulation of DNMT3B indicates increased DNA methylation activity, whereas the downregulation of H3.1 may signify changes in chromatin remodeling linked to disease progression. These findings are consistent with those of previous studies, indicating a role for DNA methylation and histone modifications in the pathogenesis of endometriosis [47]. The opposing expression patterns of DNMT1 and DNMT3B indicate complicated epigenetic reprogramming instead of homogeneous alterations in global DNA methylation [48]. DNMT1 is responsible for the maintenance of methylation, and its downregulation may result in passive hypomethylation during replication [49]. Conversely, elevated DNMT3B levels may induce de novo methylation at chromosomal locations [50]. This disparity may lead to localized hypermethylation in conjunction with global or partial hypomethylation. The redistribution of methylation marks has been documented in endometriosis and cancer, signifying dynamic and locus-specific epigenetic control. Understanding these epigenetic alterations offers valuable insights into the molecular mechanisms of endometriosis and may aid in the development of targeted epigenetic therapies for disease management.

Based on our previous bioinformatics and experimental results [19], which revealed interactions among IL-6, autophagy pathways, and miRNA networks in ovarian diseases, we suggest that post-transcriptional regulation may account for the observed disparity between IL-6 mRNA and protein levels. Specifically, potential miRNAs like miR-149 and miR-155 have been documented to regulate IL-6 translation and inflammatory signaling across many clinical situations [51]. We have broadened emphasizing these regulatory molecules as potential mediators of IL-6 suppression at the protein level. Future investigations will focus on validating these miRNAs by qRT-PCR and functional tests within the same patient group to ascertain their involvement in post-transcriptional silencing processes.

### Key Limitations of the Current Study in Ethnic Comparability

A key limitation of the current study is the absence of direct comparisons with molecular datasets from diverse ethnic populations, which constrains our capacity to definitively identify population-specific markers. This study highlights the potential heterogeneity among OE patients and the need for future studies to incorporate quantitative histological scoring systems to better capture inter-patient variability. Accordingly, intra- and inter-sample variability are important limitations and emphasize the need for larger, well-stratified cohorts in future studies. Moreover, it has been acknowledged that future studies using fresh or frozen tissues and larger sample sizes will be necessary to validate protein-level findings more comprehensively. While our research underscores specific modifications in genes like PTTG1 and DNMT3B within a Saudi cohort, variations in study design, tissue origin, analytical methodologies, and patient demographics among published studies complicate quantitative cross-ethnic comparisons. Existing datasets from Western or East Asian populations frequently differ in sample type, disease stage, and methodological techniques, constraining their direct comparability with our results. Notably, the observed IL-6 mRNA-protein discrepancy, concurrent LC3 upregulation, and the divergent DNMT1/DNMT3B expression pattern may reflect unique regulatory features, although these require validation in multi-ethnic cohorts. Consequently, whereas our findings offer a significant molecular overview of ovarian endometriosis in Saudi women, one must be cautious in ascribing these patterns solely to ethnicity. We acknowledge that the use of surgical controls may introduce potential confounding factors and that future studies with strictly inflammation-free controls are warranted. Future research using standardized multi-center cohorts, unified techniques, and extensive transcriptome or proteomic analysis will be crucial for validating and contextualizing ethnic diversity in the pathophysiology of endometriosis. Importantly, future studies will incorporate standardized semi-quantitative scoring systems to provide more robust and comparable histopathological data.

## 4. Materials and Methods

### 4.1. Study Participants and Tissue Collection

A total of 188 women were included in this study, comprising 100 control subjects and 88 patients with histologically confirmed ovarian endometriosis. The control group (a comparison group rather than ideal “healthy controls”) primarily consisted of participants who underwent total hysterectomy with bilateral salpingo-oophorectomy (TH + BSO) (94%), with the remainder undergoing cystectomy (4%) or salpingo-oophorectomy (2%). The patient cohort exhibited greater variability in surgical procedures, with oophorectomy being the most common (50%), followed by TH + BSO (34.1%), salpingo-oophorectomy (5.7%), and cystectomy (1.1%). All participants had complete clinical and surgical data (Table 1). The mean ages for the patient and control cohorts were 32.88 ± 6.63 and 33.89 ± 6.36 years, respectively. All patients and controls were claimed to be of Saudi origin and had no family history of endometriosis. The disease was confirmed histologically on formalin-fixed paraffin-embedded (FFPE) tissues.

In our study, control tissues were obtained from women undergoing hysterectomy and/or oophorectomy for non-endometriosis-related indications. Importantly, all control samples were histologically confirmed to be free of ovarian endometriosis, and cases with diagnosed endometriosis were strictly excluded. Where clinical data were available, the surgical indications primarily included benign gynecological conditions (e.g., uterine fibroids, non-inflammatory cysts, or prolapse), and no cases with known inflammatory gynecological diseases related to endometriosis were included. All control cases were confirmed to be free of ovarian endometriosis by histopathological examination. No patients with diagnosed inflammatory gynecological diseases related to endometriosis were included.

### 4.2. Sample Processing and Nucleic Acid Extraction

FFPE tissue samples were sectioned using a rotary microtome. Genomic DNA and total RNA were extracted from ovarian cyst wall/endometrioma tissue from OE patients and non-endometriotic ovarian tissues (stroma) from control subjects using a commercial kit (AllPrep DNA/RNA FFPE kit, Qiagen, Hilden, Germany). All clinical data were anonymized to protect patient privacy. The quantity and quality of the extracted DNA and RNA were assessed using a Nanodrop ND-2000C spectrophotometer (Thermo Scientific, Wilmington, DE, USA). Extracted nucleic acids were stored at −20 °C until further analysis.

We compared ovarian cyst wall/endometrioma tissue from OE patients (specifically, the cyst wall containing ectopic endometrial glands and stroma) with non-endometriotic ovarian tissues (stroma) from control subjects. These control tissues were histologically confirmed to be free of endometriosis.

### 4.3. Complementary DNA (cDNA) Synthesis

Total RNA (2000 ng/μL) was reverse-transcribed into cDNA using a high-capacity cDNA Reverse Transcription Kit (Applied Biosystems 4368814, Carlsbad, CA, USA), following the manufacturer’s protocol. The resulting cDNA solution was diluted 1:10 and stored at −20 °C.

### 4.4. RT^2^ Profiler PCR Array Analysis

Transcriptional profiling was performed using an RT^2^ Profiler array (Qiagen), which targets 84 genes associated with the P53 signaling pathway, along with five housekeeping genes (HK1-HK5). Total RNA (2.0 µg) was converted to cDNA using a First Strand Kit (4368814; Applied Biosystems, Carlsbad, CA, USA). A reaction mix was prepared by combining 102 μL of diluted cDNA with 1350 μL SYBR Green Master Mix (Qiagen) and 1248 μL of RNase-free water, yielding a final volume of 2700 μL. Twenty-five microliters of the reaction mix were added to each well of the array. The array also included controls for genomic DNA contamination, reverse transcription, and positive PCR. The reaction was run on an Applied Biosystems Quant Studio 5 Real-Time PCR System with cycling parameters set to a 10-min pre-incubation at 95 °C, followed by 45 cycles of 15 s at 95 °C and 60 s at 60 °C with fluorescence data acquisition.

### 4.5. Quantitative Real-Time PCR (qRT-PCR)

For the gene expression experiments, we analyzed tissues from at least 15–20 independent patients per group. Optimum amplification for individual gene targets was achieved via qRT-PCR. Primers were manually designed to yield amplicons no greater than 120 bp, with a minimum length of 18 nucleotides and optimal GC content to minimize secondary structures. Primer specificity was confirmed via a BLAST search. Oligonucleotide primers (Supplementary Materials S1) were synthesized by Macrogen, Inc. (Seoul, Republic of Korea). qRT-PCR was performed using a Universal SYBR Green Supermix (1725120, Bio-Rad, Hercules, CA, USA). Each 96-well plate contained a 10 μL reaction mix: 4 μL SYBR Green Supermix, 1 μL ROX, 2 μL of cDNA, 1 μL of both forward and reverse primers (10 pmol stock concentration), and 2 μL of nuclease-free water. Reactions were performed in triplicate on a QuantStudio™ 7 Flex Real-time PCR System (Applied Biosystems). GAPDH was used as the reference gene for data normalization.

### 4.6. Immunofluorescence Staining and Confocal Microscopy

For immunofluorescence, we used n = 10 per group (10 healthy patients and at least 10 OE patients). Slides were deparaffinized, rehydrated, and rinsed with phosphate-buffered saline (PBS). Antigen retrieval was performed using a solution of 0.1% sodium citrate and 0.1% Triton X-100. Non-specific staining was blocked with a blocking solution for 45 min at room temperature. Primary antibodies were incubated overnight at 4 °C. Following four washes with 1× PBS, corresponding secondary antibodies were incubated for 45 min at room temperature in the dark. Nuclei were counterstained with Hoechst solution (1:30,000 dilution). Slides were mounted with 50% glycerol/TE solution and sealed. Image acquisition was performed using a spinning disk confocal microscope (Zeiss, Oberkochen, Germany). Signal intensity was analyzed using Zen 3.1 service (ZEN lite) and quantified with GraphPad Prism Software (version 10.2).

### 4.7. Western Blot Analysis

For the Western blot, we used n = 10 per group (10 healthy patients and at least 10 OE patients). Protein extraction from FFPE tissues, which presents a challenge due to formalin-induced cross-linking, was performed using a Minute Protein Extraction Kit (Invent Biotechnologies, FE-025UK, Plymouth, MN, USA). Thin tissue sections were deparaffinized and homogenized in Buffer B. Following centrifugation, the supernatant was collected, and protein concentration was determined using a Bradford assay. Twenty micrograms of protein were resolved via SDS-PAGE and transferred onto polyvinylidene fluoride membranes (Merck Millipore, Burlington, MA, USA). Membranes were blocked with fetal bovine serum (FBS) blocking solution for 1 h, followed by overnight incubation at 4 °C with primary antibodies against β-actin (Abcam, Cambridge, UK; ab 1801; dilution 1:1000),LC3((Abcam, Cambridge, UK; ab229327; dilution 1:1000) and IL6 (Abcam, Cambridge, UK; ab 9324; dilution 1:500). After washing, membranes were incubated with horseradish peroxidase-linked secondary antibodies (1:3000) for two hours. Chemiluminescent signals were captured using an ECL clarity buffer and a ChemiDoc MP Imaging System (Bio-Rad, USA).

### 4.8. Statistical Analysis

Statistical analyses were conducted using the SPSS software package (version 20.0; SPSS Inc., Chicago, IL, USA). The Chi-square (χ^2^) test was applied to determine *p*-values between the patient and control groups. Gene expression levels were compared using GraphPad Prism software (version 10.2). Data distribution was assessed for normality prior to analysis, and appropriate statistical tests were applied accordingly. For comparisons between two groups, we used an unpaired two-tailed Student’s *t*-test for normally distributed data, while non-parametric alternatives were considered where applicable. A *p*-value less than 0.05 was considered statistically significant.

### 4.9. Ethical Approval

This study was approved by the Ethical Committee and Institutional Review Board of the College of Medicine Research Center at King Saud University Medical City (reference number E-22-6750).

## 5. Conclusions

The findings of this study offer significant insights into the molecular mechanisms of ovarian endometriosis. Histopathological changes that alter gene expression, modulate inflammatory cytokines, increase autophagic activity, and induce epigenetic modifications collectively contribute to the persistence and progression of endometriotic lesions and associated infertility. Focusing on critical pathways related to inflammation, autophagy, and epigenetic regulation could provide innovative therapeutic approaches for the management of endometriosis. Future research should focus on clarifying the specific molecular mechanisms that control IL-6 dysregulation, the function of autophagy in the survival of endometriotic cells, and the influence of epigenetic modifications on disease progression. Furthermore, exploring potential biomarkers for early diagnosis and developing targeted therapies based on gene expression and epigenetic modifications may enhance clinical outcomes in patients with ovarian endometriosis.

## Figures and Tables

**Table 1:** Descriptive statistics (mean, standard deviation, and standard error of the mean) for age and body mass index (BMI) in control and patient groups.

Group	*N*	Mean	Std. Deviation	Std. Error Mean
Age Control Patient	95	32.88	±6.63	≈0.68
	88	33.89	±6.36	≈0.681
BMI Control Patient	93	32.91	7.868	≈0.816
	74	27.50	6.019	≈0.700

## Data Availability

The original contributions presented in this study are included in the article/Supplementary Materials. Further inquiries can be directed to the corresponding author.

## Supplementary Materials

The following supporting information can be downloaded at: https://www.mdpi.com/article/10.3390/ijms27104598/s1.
