# Supplementary material for: Molecular Characterization of Ovarian Endometriosis in Saudi Arabian Women: Insights into Inflammatory, Autophagic, and Epigenetic Dysregulation
Source: Int J Mol Sci. 2026 May 20;27(10):4598. doi: 10.3390/ijms27104598 (PMC13206791; doi:10.3390/ijms27104598)
Supplement: Supplementary file 1 [file ijms-27-04598-s001.zip › ijms-4256014-supplementary.pdf]

S1. List of primers used in qRT-PCR.

Gene symbol	Primer category	Primer Sequence (5'----3')	Product size
**GAPDH**	Forward<br>Reverse	CTCAGACACCATGGAAGG<br>AGTTAAAAGCAGCCCTGGTGA	95
**IL10**	Forward<br>Reverse	CCTGGGGGAGAACCTGAAGA<br>CACGGCCTTGCTCTTGTTTT	85 bp
**Histone**	Forward<br>Reverse	GTGTTCCGCTGTGCTGTTTT<br>GCCTTGCCACCAGTAGACTT	73 bp
**IL6**	Forward<br>Reverse	GAGAAGCCCTTCTCCTGTCCCC<br>TGCCCTCAAGATGCACATCCGA	85 bp
**ESR1**	Forward<br>Reverse	GCTTACTGACCAACCTGGCAGA<br>GGATCTCTAGCCAGGCACATTC	100 bp
**ESR2**	Forward<br>Reverse	ATGGAGTCTGGTCGTGTGAAGG<br>TAACACTTCCGAAGTCGGCAGG	100 bp
**CDK1**	Forward<br>Reverse	GGAAACCAGGAAGCCTAGCATC<br>GGATGATTCAGTGCCATTTTGCC	90 bp
**CDKN1**	Forward<br>Reverse	AGGTGGACCTGGAGACTCTCAG<br>TCCTCTTGGAGAAGATCAGCCG	90 bp
**DNMT1**	Forward<br>Reverse	AGGTGGAGAGTTATGACGAGGC<br>GGTAGAATGCCTGATGGTCTGC	84pb
**LC3**	Forward<br>Reverse	CATCTGCCCCTCACCCAC<br>CAGGGGCCAGTCTTTCAGG	71bp
